# Comparison of mutation landscapes of pretreatment versus recurrent squamous cell carcinoma of the oral cavity: The possible mechanism of resistance to standard treatment

**DOI:** 10.1002/cnr2.2004

**Published:** 2024-03-13

**Authors:** Tongchai Payungwong, Krittaya Angkulkrerkkrai, Amphun Chaiboonchoe, Wirote Lausoontornsiri, Siwanon Jirawatnotai, Somjin Chindavijak

**Affiliations:** ^1^ Siriraj Center of Research Excellence in Systems Pharmacology, Department of Pharmacology, Faculty of Medicine Siriraj Hospital Mahidol University Bangkok Thailand; ^2^ Center of Excellence of Otolaryngology Head and Neck Surgery Rajavithi Hospital Bangkok Thailand; ^3^ Medical Oncology Samitivej Hospital Bangkok Thailand

**Keywords:** cancer cell cycle, DNA repair, epigenetics, mutation landscape, recurrent oral squamous cell carcinoma

## Abstract

**Background:**

A high recurrent rate of oral squamous cell carcinoma (OSCC) is a major concern in head and neck cancer treatment. The study of the genetic mutation landscape in recurrent OSCC may provide information on certain mutations associated with the pathobiology and treatment response of the OSCC.

**Aim:**

We investigated the mutation landscape of matched pretreatment and recurrent tumors to understand the influence of genetic mutations on the pathobiology and clinical outcomes in OSCC.

**Methods and Results:**

We sequenced 33 formalin‐fixed paraffin‐embedded (FFPE) recurrent tumors, primary tumors, and primary tumors before recurrence that matched the recurrent tumors collected from Rajavithi Hospital during 2019–2021. We identified recurrent mutations from these samples by the Oncomine Ion Torrent‐based next‐generation sequencing on the 517 cancer‐associated gene panel. From the results, we found that the most commonly mutated gene in the cohort is a histone methyltransferase *KMT2D* (54.55%), implicating that aberrance in epigenetic regulation may play a role in oral cancer tumorigenesis. Functional protein association network analysis of the gene frequently mutated in the recurrent tumors showed enrichment of genes that regulate the cancer cell cycle, that is, *MRE11A, CDKN2A*, and *CYLD*. This finding was confirmed in the primary‐recurring matched pair. We found that recurrent tumors possess a small but recurring group of genes, with presumably the subclonal mutations driving the recurrence of the tumor, suggesting that the recurrent disease originated from a small fraction of the cancer cell that survives standard treatment. These genes were absent in the primary tumor with a good response to the standard treatment. On the other hand, we found an enrichment of DNA repair genes, namely *ATR*, *BRCA1*, *BRCA2*, *RAD50*, and *MUTYH*, in nonrecurrent tumors suggesting that the mutations in the DNA repair pathway may at least partially explain the different response to the standard treatment.

**Conclusions:**

Our pilot study identified pathways of carcinogenesis in oral cancer and specific gene sets that indicate treatment responses and prognoses in this group of patients.

## 1 INTRODUCTION

Oral squamous cell carcinoma (OSCC) is a prevalent type of head and neck cancer.^1^, ^2^ In 2020, the worldwide incidence of new OSCC cases was 377 713 cases, and the number of deaths from OSCC was 177 757.^3^ The major risk factors for OSCC are smoking, alcohol consumption, and human papillomavirus.^4^, ^5^ While surgical therapy followed by radiotherapy or radio‐chemotherapy is the standard treatment for OSCC,^6^ recurrence after treatment can occur, ranging from 20% up to 86%.^7^, ^8^ The recurrence of OSCC is a major problem for treatment and can result in poor survival rates.^9^ The recurrence rate of OSCC is high, following standard treatment: approximately 40%–60% in advanced stages^10^, ^11^, ^12^, ^13^ and 10%–25% in earlier stages.^14^, ^15^ Recurrence of OSCC is usually difficult to treat due to the limited treatment options available, and these recurrent tumors represent the progeny of resistant cancer cells that successfully evaded standard treatment.^16^ Moreover, there are currently no predictive biomarkers for the recurrence after standard treatment of OSCC. Therefore, studying the detailed genetic mechanism of recurrence after standard treatment in OSCC is essential to guide future clinical practices.

The tumorigenesis of OSCC is believed to be driven by specific genetic alterations. Therefore, understanding the genetic alteration landscape in OSCC can aid in the development of precision medicine. However, there is limited knowledge and research investigating the mechanism of recurrence after therapeutic treatment in OSCC for improving outcomes.^17^, ^18^, ^19^, ^20^ There are a number of reports on the genetic mutation landscape of OSCC. Previous reports of the snapshot OSCC mutation landscape revealed *TP53*, *CDKN2A*, *PIK3CA*, *HRAS*, *NOTCH1*, *CHUK*, and *ELAVLI* as frequently mutated genes.^19^, ^21^, ^22^ However, only a few studies focused on the relative genetic information between primary and recurrent OSCC tumors. Although ideally, matched primary and recurrent OSCC tumors from the same patients should be compared for the highest quality data, very few such data exist.

To understand the dynamic change of genetic mutations that are influenced by standard treatment, a study with a small number of patients on mutation landscapes in recurrent and metastatic head and neck cancer was performed.^23^ The result revealed that mutations of *C17orf104*, *ITR3*, and *DDR2* were specifically found in the recurrent or metastatic tumor but not in the primary. This result is suggesting that the activation of particular genes may facilitate head and neck cancer recurrence.

In this study, we focus on OSCC and aim to identify mutated genes that may contribute to recurrence after standard treatment by comparing gene mutations from FFPE tissue samples of recurrent OSCC before and after standard treatment, a portion of which are paired, matched tumors. We also studied mutations in OSCC with good response to the standard therapy relative to recurrent tumors, hoping to identify mutated genes that may confer sensitivity to standard treatment by studying genes that were correlated with complete response compared with genes that correlated to recurrence.

## 2 METHODS

### 2.1 Sample collection and inclusion criteria

The retrospective study protocol and archival FFPE samples analyzed were approved by the Rajavithi ethic committee (EC No.64102) and Siriraj Institutional Board (SIRB No. 104/2564[IRB1], COA no. Si 344/2021), whereby the anonymized archival FFPE samples were provided to the researchers without the requirement for patient consent. Naïve oral cancer patients who underwent surgery, followed by standard treatment during 2019–2021 in Rajavithi Hospital, were included in this study. The recurrent group samples included FFPE from primary oral cancer patients who had regular follow‐ups in the clinic with evidence of recurrence within 24 months. Of these samples, 7 cases have matching primary and recurrent FFPE blocks. The nonrecurrent control group included FFPE in oral cancer patients who had regular follow‐ups in the clinic with no evidence of recurrence for at least 36 months and found 9 cases. The patients who were lost to follow‐up or had no pathological report of recurrence were excluded from the trial.

### 2.2 Next‐generation sequencing of OSCC

The genomic DNA was extracted from FFPE by using MagMAX™ Cell‐Free Total Nucleic Acid Isolation Kit (ThermoFisher Scientific). Qubit quantification was performed after DNA extraction. TaqMan™ GUSB gene assay was performed as a proxy determination of the amplifiable FFPE DNA. Deaminated cytosine bases, commonly found in FFPE specimens, were enzymatically removed by treatment with uracil DNA glycosylase (ThermoFisher Scientific).Target sequencing libraries were constructed with Oncomine™ Comprehensive Assay Plus (DNA+RNA) (ThermoFisher Scientific) with a target 517 cancer‐associated gene panel. Then, target sequencing was performed by Ion GeneStudio™ S5 System (ThermoFisher Scientific) following the manufacturer's instructions.

### 2.3 Next‐generation sequencing analysis

The sequencing data were uploaded to Ion Reporter software (V. 5.18) (ThermoFisher Scientific) and analyzed by Oncomine Comprehensive Plus w2.3 DNA‐Single sample workflow using human genome assembly GRCh37(hg 19) as a reference for alignment. The abundance of FFPE artifacts was assessed by the software via three parameters, that is, deamination score above 60, the signature pattern of somatic mutation compatible with deamination of 5‐methylcytosine, UV damage, or FFPE processing, and abnormally high tumor mutation burden (above 10 muts/Mb). If one of the criteria is met, FFPE artifacts were filtered using a variant allele frequency (VAF) cutoff 10%. The sequencing quality was assessed by the software via four measurements, that is, mapped read >22 million, mean depth >800×, percent uniformity >80%, and percent on target >85%. Gene annotation was applied by the Oncomine Comprehensive Plus Annotations v1.2. Minor allele frequency was filtered using the Database for Single Nucleotide Polymorphisms (dbsnp v. 154) and 5000Exomes (V. 20 161 108).

Somatic variant calling was performed by applying the filter chain “Oncomine™ extended 5.18v2,” which essentially selected only the somatic the Catalogue Of Somatic Mutations In Cancer variants. The results then were manually analyzed for only likely pathogenic or pathogenic variants according to ClinVar (V. 20201121), with a sequence coverage of at least 100×.

### 2.4 Protein–protein interaction networks functional enrichment analysis

The pathway or protein–protein interaction networks that may involve the pathogenesis of OSCC was determined by a search tool for retrieval of interacting genes (STRING) database.^24^ The lists of the gene identified to be unique to the recurrent, and the non‐recurrent tumors were applied to the STRING tool with the active interaction sources set to include the data from experiments, databases, and co‐expression (only Homo sapiens specified) to construct the protein–protein interaction networks.

## 3 RESULTS

### 3.1 Patient characteristics

We performed an investigation in 33 FFPE blocks from 26 patients with 17 cases of recurrent and 9 nonrecurrent OSCC cases. The patient's characteristics were typical of primary curative surgery, followed by standard treatment for OSCC patients, summarized in Table 1. Briefly, the average age of OSCC patients is 58 years old, 18 of 26 cases had a history of substantial tobacco exposure, and 19 of 26 cases with a history of alcohol use. Ten of 26 (38%) were cancer of the tongue cases. The adjuvant treatment received was postoperative radiotherapy alone (14 of 26 cases), chemo‐radiotherapy (7 of 26 cases), chemotherapy alone (1 of 26 cases), and no treatment (4 of 26 cases), as shown in Figure S1A. In the nonrecurring case, most patients presented with tumors in the early stages. In contrast, recurrent OSCC patients usually present with tumors in the advanced stages with or without cervical lymph node involvement as shown in Figure S1B,C. The median relapse‐free survival in the recurrent group was 10 months.

**TABLE 1:** Patient characteristics.

Characteristics	All patient *N* = 26	Nonrecurrent patient *N* = 9	Recurrent patient *N* = 17
	*N*	*N*	*N*
Average age (years)	58	58	60
Gender			
Male	20	9	11
Female	6	0	6
Smoking status			
Never	8	3	5
Former	7	3	4
Active	11	3	8
Unknown	0	0	0
Alcohol consumption			
Never	7	1	6
Former	8	6	2
Active	11	2	9
Unknown	0	0	0
Stage of tumor (*T*)			
I	4	4	0
II	8	5	3
III	4	0	4
IV	10	0	10
Stage of tumor (*N*)			
0	12	6	6
I	2	1	1
II	12	2	10
Location of tumor			
Tongue	10	5	5
Floor of mouth	7	4	3
Buccal	1	0	1
Alveolar ridge	6	0	6
Hard palate	2	0	2
Other	0	0	0
Postoperative treatment			
Radiotherapy	14	3	11
Chemo‐radiotherapy	7	2	5
Chemotherapy	1	1	0
No	4	3	1
Average time from tumor diagnosis to recurrence diagnosis (months)	10	0	10

### 3.2 KMT2D may play role in oral cancer tumorigenesis

**FIGURE 1:**
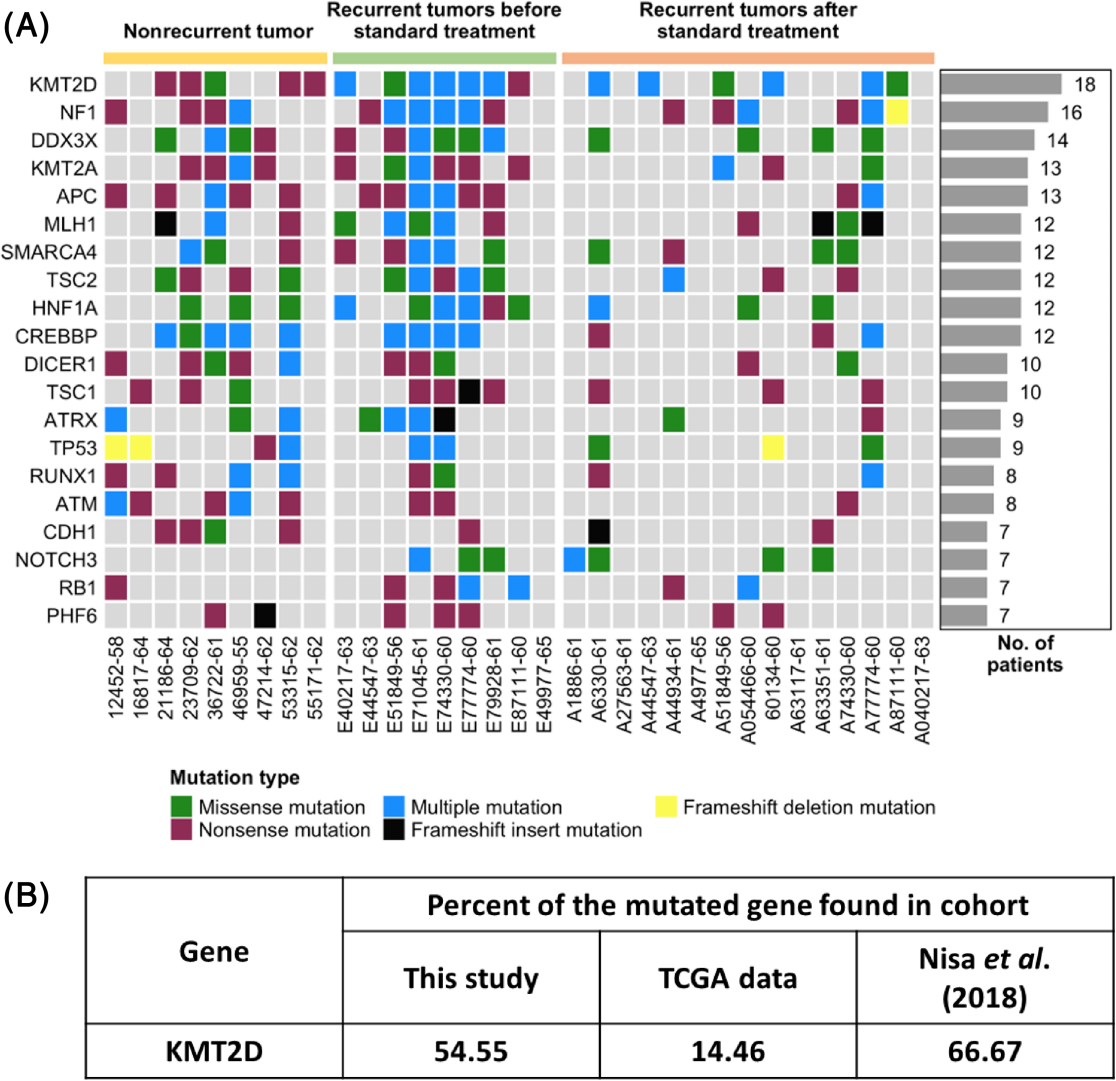
Mutation landscape of oral squamous cell carcinoma (OSCC) patients in this study. (A). Heatmap of top 20 frequently mutated genes. Each row indicates the gene and each column indicates the patient. The bars on the right‐hand side show numbers of patients containing mutated genes in each row. Different colors presenting types of mutation shown at the bottom of the figure. Colored bars on top of the figure indicate groups of OSCC patients. (B). Percents of mutated KMT2D from three studies.

For all samples, we opted for targeted deep sequencing of 517 cancer‐associated genes, which divided the results into three groups, the primary tumor which did not recur, the primary tumor before recurrence, and recurrent tumors post‐standard treatment. The sequencing results were supplied in the Supplementary Information. The resulting mutations were shown in Figure S2 with the TP53 gene as a top mutated gene. The mutations representing the pathogenic and likely pathogenic genes in our cohort were selected as described. The resulting mutation landscape was shown in Figure 1A and Figure S3. Our data revealed that the *KMT2D* mutation, which is an epigenetic gene, is the most commonly found gene among all three groups. The mutation rate of the *KMT2D* was 54.55%. A similar finding was also found in The Cancer Genome Atlas (TCGA), and in the study by Nisa and colleagues^25^ in 2018, in which the *KMT2D* mutations were 14.46% and 66.67%, respectively (Figure 1B). Our study confirmed that *KMT2D* may play a role in oral cancer tumorigenesis but may not be the ideal marker for disease prognosis.

### 3.3 Mutations in genes regulating cancer cell cycle and differentiation may confer the recurrence of OSCC after completed treatment

**FIGURE 2:**
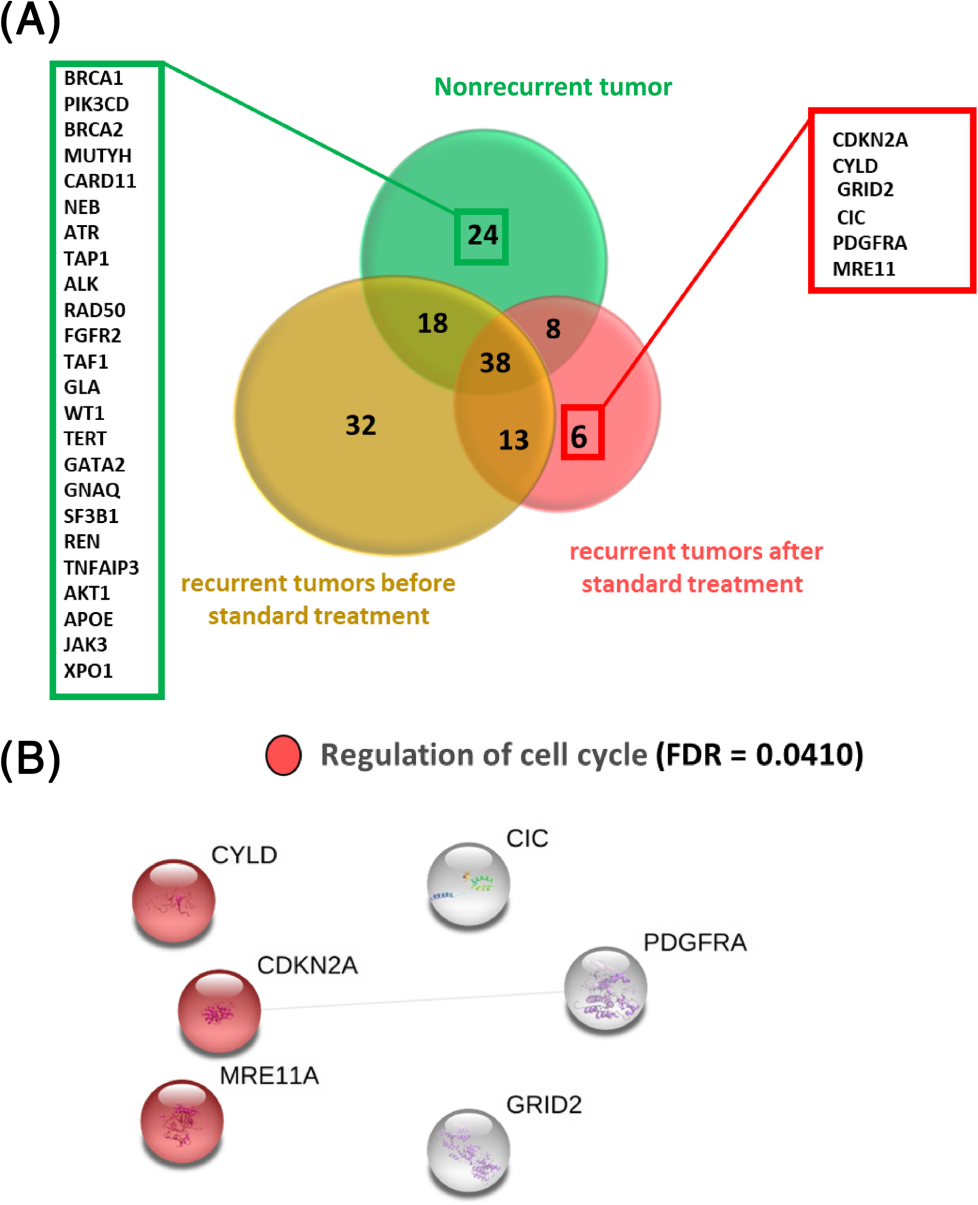
Sets of unique genes found in each group of oral squamous cell carcinoma (OSCC) sample. **(**A) Venn diagram shows sets of unique genes found in this study. Different colors in the Venn diagram represent the groups of OSCC sample, nonrecurrent tumor (green), as a recurrent tumor before standard treatment (orange), and recurrent tumor after standard treatment (red). Red box shows the set of unique genes only found in a recurrent tumor after standard treatment and green box shows the set of unique genes only found in a nonrecurrent tumor. (B). Genes uniquely found in recurrent tumors after standard treatment are genes in cell cycle regulation. Lines connected between genes indicate functional or physical interactions between these genes based on search tool for retrieval of interacting genes.

**FIGURE 3:**
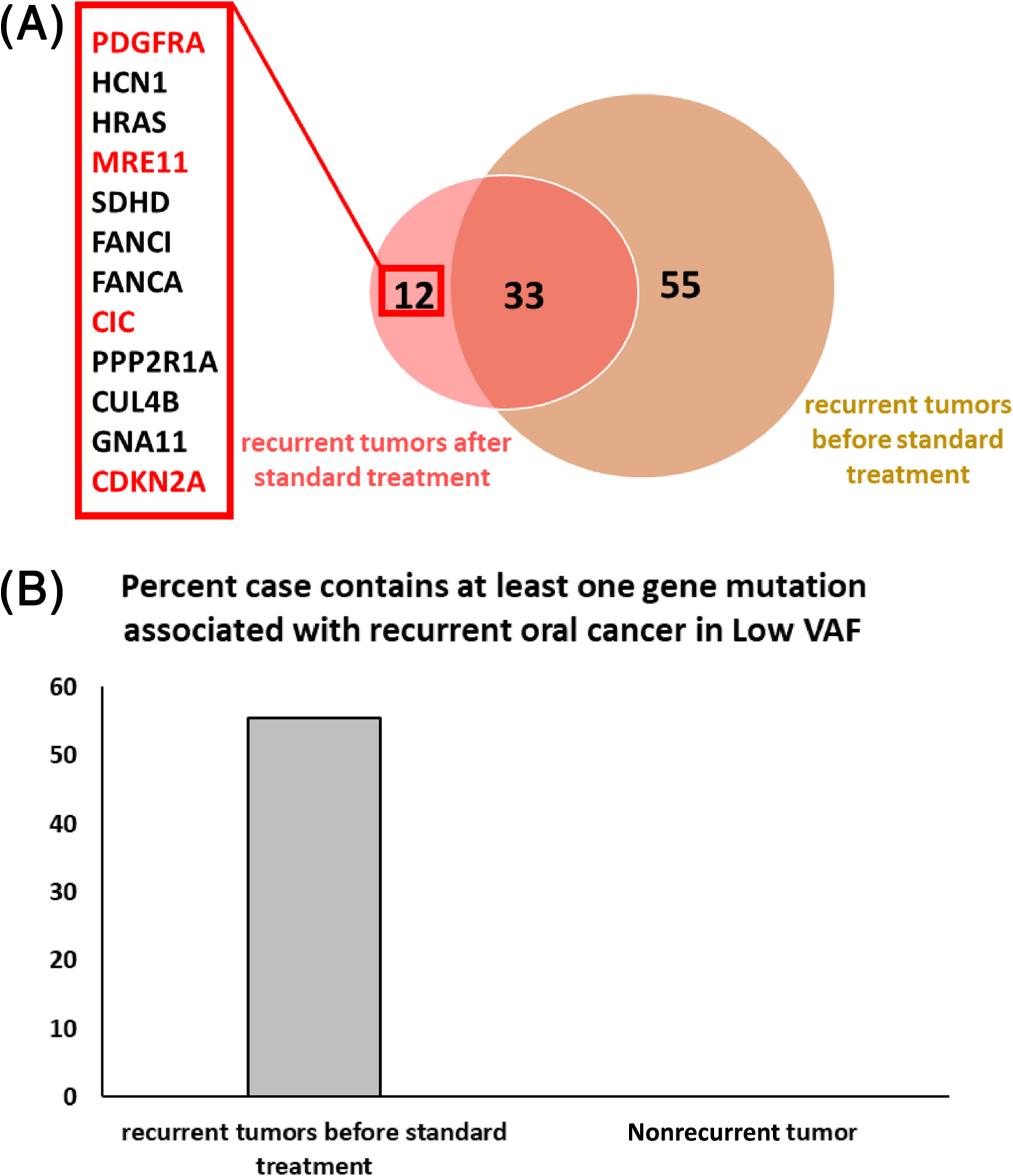
Enrichment of cell cycle regulation gene in pair‐matched samples. (A). Venn diagram of the pair‐matched tumor shows a set of unique genes in cell cycle regulation. Brown color indicates recurrent tumors before standard treatment and red color indicates recurrent tumors after standard treatment. Red box shows the set of unique genes only found in a recurrent tumor after standard treatment. The text with red color shows the unique genes that regulate the cell cycle. (B). Bar graphs of the percent of cases containing at least one cell cycle regulation gene (≥5% variant allele frequency [VAF]).

To identify gene mutations that may contribute to a recurrence of OSCC, the three groups of OSCC patients, gene mutation frequencies were compared as shown in Figure 2A. Of all the gene mutations detected, a total of six (*CDKN2A*, *CYLD*, *MER11A*, *CIC*, *GRID2*, and *PDGFRA*), were uniquely associated with tumor recurrences. To identify potential pathways of enrichment for these mutated genes, we used STRING to identify gene pathways and the results showed enrichments of pathways involved with cancer mutant cell cycle and differentiation (Figure 2B). To establish how important these mutated genes are (Figure 2A,B), we compared the mutated genes in a group of recurrent OSCC patients, who have paired match tumors before and after treatment as shown in Figure 3A. The results confirmed that the gene identified in Figure 2A,B was also found in the match‐paired recurrent tumors after treatment. These results suggest that the mutations in cancer cell‐cycle regulation/differentiation genes may play an important major role in the recurrence of OSCC after standard treatment is completed.

### 3.4 The recurrent disease may be rare clones of OSCC that survive during treatment

Figures 1A and 2A show that the number of mutant genes found in recurrent tumors is less than in the nonrecurrent and primary tumors before relapse. This result may come from a small fraction of OSCC that survive standard treatment; hence, the treatment may allow the survival of the cells containing these mutations. To investigate this hypothesis, we searched and found the genes identified from Figure 2A in the VCF files in both nonrecurrent and primary tumors before relapse with VAF ≤5%. Interestingly, this set of the genes that correlated with recurrence was specifically found in primary tumors before relapse, but never found in the nonrecurrent tumors (56% compared with 0% of cases containing at least one gene mutation associated with recurrent oral cancer in VAF ≤5%) (Figure 3B). This result suggests that recurrent OSCC originated from a small fraction of OSCC that survived initial treatment.

### 3.5 Mutations in DNA repair genes may confer nonrecurrent cases

Figure 2A showed the result of 24 mutated genes, which were found only in nonrecurrent tumors. To identify the pathway of enrichment for these mutated genes, STRING was used to analyze proteins in the same complex. We found enrichment of DNA repair genes (*ATR*, *BRCA1*, *BRCA2*, *RAD50*, *and MUTYH*) as shown in Figure 3A. These mutated genes, especially the mutated *BRCA1*, *ATR*, *and RAD50* genes, were found in more than one nonrecurrent tumor (22.22%) (Figure 2B). We also identified mutated DNA repair genes in all three groups of our OSCC and found that 55.6% of cases with a gene mutation in the DNA repair pathway can be found in nonrecurrent tumors. In contrast, only 0% of cases with a gene mutation in the DNA repair pathway can be found in primary tumors recurrent tumors, respectively. This result suggests that mutant DNA repair genes may be a unique characteristic of tumors that do not recur.

## 4 DISCUSSION

To elucidate the mutational profiles of OSCC that may contribute to drug response, we set out to perform next generation sequencing on a cohort containing primary versus recurrent tumors, some of which matched‐pair tissue samples. We found that the most common mutation found in our OSCC study was *KMT2D*, which is also reported to be one of the most common mutations in the TCGA cohort and again in another previous study.^25^ Mutation of *KMT2D* was also common in other types of cancer, such as small‐cell lung cancer and pheochromocytoma.^26^, ^27^ The role of *KMT2D* mutation in tumorigenesis was studied by Maitituoheti and colleagues in 2020 who found that *KMT2D*‐deficient melanoma cells were associated with an epigenetic change in H3K4me1‐marked enhancer activtion.^28^ While a further study of the *KMT2D* function in the epigenetic and tumorigenesis of OSCC is still required, our finding suggests that *KMT2D*‐mediated epigenetic changes may play an important role in OSCC tumorigenesis.

Our study identified a set of unique genes in recurrent tumors, which have three genes that may contribute to a recurrence factor in our recurrent OSCC. These were *CDKN2A*, *MRE11A*, and *CYLD*.*CDKN2A* (p16) gene has been well studied in head and neck cancer including OSCC^18^, ^29^, ^30^ and it shows the correlation with loss‐of‐function *CDKN2A* in the major recurrence of hand‐neck cancer.^31^, ^32^ By analyzing a set of unique genes in recurrent tumors using STRING, we identified the deregulation of the cell cycle pathway, which appears to be closely associated with recurrence in OSCC. This finding was supported by a report on the function of *CDKN2A* and *CYLD*.

*CDKN2A* gene functions lead to cell cycle arrests by inhibiting the function of CDK4 protein.^33^ Several independent studies demonstrated that loss‐of‐function *CDKN2A* in oral cancer is correlated with worsened prognoses.^34^, ^35^, ^36^ Interestingly, data from a mouse model with *cdkn2a* ^*−/−*^ genotype showed that *cdkn2a* loss of function is a rate‐limiting step in oral cancer formation^37^ and targeting the CDK4/CDKN1A/RB1 pathway may be an attractive strategy to treat OSCC.^38^ *CYLD* also can regulate cell‐cycle progression by inactivating HDAC6 and increasing the levels of acetylated tubulin.^39^ Loss of function mutation of *CYLD* has been shown to be associated with cisplatin resistance in oral cancer.^40^ In the same publication, suppression of CYLD by *CYLD*‐specific siRNA can reverse the cisplatin‐resistant phenotype, indicating that *CYLD* is one of the major proteins that facilitate oral cancer resistance. Even with the limited number of patients in this cohort, our analyses were informative and precise in identifying the major genes known to confer resistance to OSCC. Our study also pointed out that the deregulation of the cell cycle regulatory proteins may be a key event that facilitates OSCC resistance to standard therapy. Therefore, these findings highlight small molecule cell cycle inhibitors as a potential therapeutic target for recurrent OSCC.^41^, ^42^

Heterogeneity and clonal selection may also be driving the recurrence in OSCC.^43^ Our results shown in Figure 3B also suggest that standard treatment induces subclonal selection pressure. This is because, we found a set of unique genes in recurrent tumors preexisting in the primary tumor before the treatment as a subclonal population. Some studies on OSCC show the clonal evolution from subclonal types in primary tumor.^43^, ^44^ These preexisting genes in recurrent tumors, also detected in the primary tumor, were previously studied in breast cancer.^33^ The study demonstrated that there are subclonal populations of breast cancer cells that contain a mutation landscape that promotes resistance to chemotherapy.^45^

**FIGURE 4:**
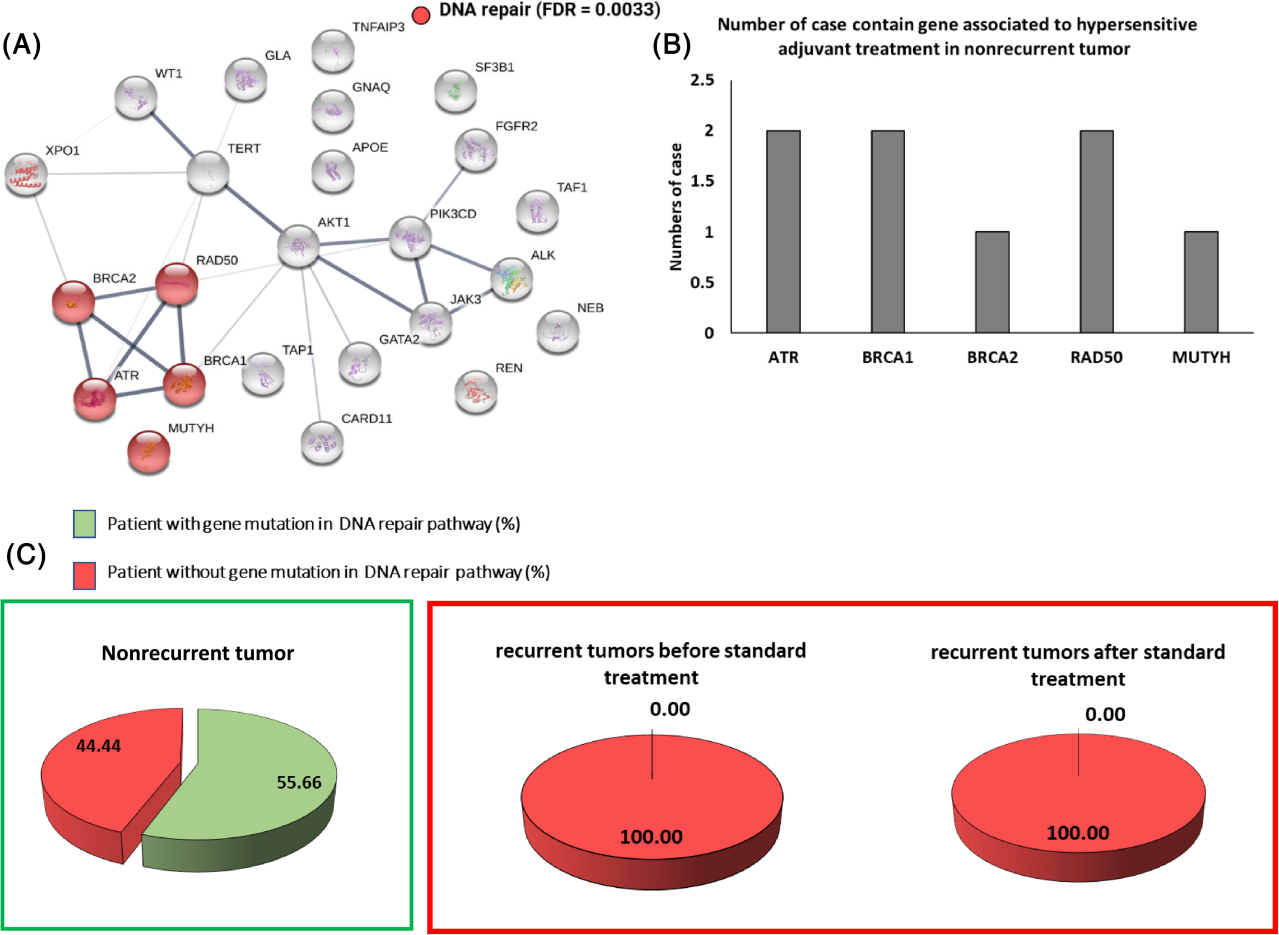
Mutations DNA repair genes may confer sensitivity to the standard treatment. (A) Enrichment of genes in DNA repair pathway. The set of genes uniquely found in nonrecurrent tumors. Lines connected between genes in the DNA repair pathway indicate functional or physical interactions between these genes based on search tool for retrieval of interacting genes. (B) Bar graphs show the numbers of cases containing mutations in DNA repair genes only found in nonrecurrent tumors. (C) Percents of patients with and without mutations genes in DNA repair pathway.

The unique finding of DNA repair gene mutations in tumors with a good response to radiotherapy or chemo‐radiotherapy was supported in a previous study in a mouse model of engrafted *BRCA1*‐deficient tumors.The mouse models showed hypersensitivity to radiation.^46^ The finding of mutant DNA repair genes may be one factor to indicate one of the prediction methods for patients who may benefit from standard treatment as shown in Figure 4C. In agreement with our result, cancer cells containing DNA repair gene defects were shown to be hypersensitive to platinum‐based chemotherapy, such as cisplatin.^47^ This may explain the typically good responses to DNA damage‐induced first‐line therapies in this disease. It is also interesting to see, whether PARP inhibitors, such as olaparib and rucaparib, which are currently used in ovarian and breast cancer with DNA repair defects,^48^ may be also used in OSCC containing mutations of DNA repair genes. From these data, it appears to be a plausible conclusion that the use of data on DNA repair gene mutations may help to guide treatment plans for primary OSCC as well as the follow‐up after the completion of each treatment. On the other hand, the ability to avoid unnecessary treatments with low probability of treatment success may improve patient quality of life. To confirm the findings of this study, we plan to expand our cohort by collecting more samples and applying a new molecular technology to identify the molecular changes in this cancer.

In conclusion, our study found a strong relation between the high rate of KMT2D mutation and OSCC, suggesting role of epigenetics in OSCC tumorigenesis. We also found evidence of preexisting subclones in the primary tumor, staying dormant in the pretreatment tumors of our recurrent OSCC group. A set of unique genes that regulate the DNA repair pathway in the tumors will indicate a good outcome after completed treatment. This pilot study may also facilitate us to conduct a new model clinical trial for molecular‐guided therapy in the future.

## AUTHOR CONTRIBUTIONS

**Tongchai Payungwong:** Conceptualization (lead); data curation (lead); formal analysis (lead); methodology (lead); software (lead); visualization (lead); writing – original draft (lead); writing – review and editing (supporting). **Krittaya Angkulkrerkkrai:** Data curation (supporting); resources (supporting). **Amphun Chaiboonchoe:** Formal analysis (supporting); visualization (supporting). **Wirote Lausoontornsiri:** Conceptualization (supporting); formal analysis (supporting); investigation (supporting); methodology (supporting); visualization (supporting); writing – original draft (supporting); writing – review and editing (supporting). **Siwanon Jirawatnotai:** Conceptualization (supporting); data curation (supporting); formal analysis (supporting); funding acquisition (lead); investigation (supporting); methodology (supporting); visualization (supporting); writing – original draft (supporting); writing – review and editing (supporting). **Somjin Chindavijak:** Conceptualization (supporting); data curation (supporting); formal analysis (supporting); investigation (lead); methodology (supporting); project administration (lead); resources (lead); supervision (lead); writing – original draft (supporting); writing – review and editing (lead).

## CONFLICT OF INTEREST STATEMENT

The authors have stated explicitly that there are no conflicts of interest in connection with this article.

## ETHICS STATEMENT

All procedures met the Helsinki Declaration of the World Medical Association ethical standards. The study protocol and archival formalin‐fixed paraffin‐embedded samples analyzed were approved by the Rajavithi ethic committee (EC No.64102) and Siriraj Institutional Board (SIRB No. 104/2564[IRB1], COA no. Si 344/2021). Patient consent was waived by Rajavithi and Siriraj IRBs as the Boards found that this research meets the requirements for a waiver of consent under 45 CFR 46.116 and 46.117; 21CFR 50.23 and 50.24; 21 CFR 56.109, since the study will use existing data/sample collection.

## Supporting information


**Figure S1.** Pie charts of patient characteristics in our cohort. (A) Postoperative treatment data. (B) Comparison of Stage of tumor (*T*) between nonrecurrent patients and recurrent patients. (C) Comparison of stage of tumor (*N*) between nonrecurrent patients and recurrent patients. The numbers in the pie charts represent the number of cases.

**Figure S2.** Mutations in the OSCC patients in this study. (A) variant classification in the OSCC, *Y*‐axis indicates types of variant and *X*‐axis indicates numbers of variant. (B) Top 10 frequently mutated genes, *Y*‐axis indicates genes and *X*‐axis indicates numbers of variant.

**Figure S3.** Mutation landscape of OSCC patients in this study. Each row indicates the gene, and each column indicates the patient. The bars on the right‐hand side show the number of patients containing mutated genes in each row. Types of mutation are indicated in colors at the bottom of the figure. Colored bars on top of the figure indicate the group of OSCC patients.

## Data Availability

The data that support the findings of this study are available from the corresponding author upon reasonable request.
