# Supplementary material for: Comparison of mutation landscapes of pretreatment versus recurrent squamous cell carcinoma of the oral cavity: The possible mechanism of resistance to standard treatment
Source: Cancer Rep (Hoboken). 2024 Mar 13;7(3):e2004. doi: 10.1002/cnr2.2004 (PMC10935893; doi:10.1002/cnr2.2004)
Supplement: Supplementary file 1 — Figure S1. Pie charts of patient characteristics in our cohort. (A) Postoperative treatment data. (B) Comparison of Stage of tumor (T) between nonrecurrent patients and recurrent patients. (C) Comparison of stage of tumor (N) between nonrecurrent patients and recurrent patients. The numbers in the pie charts represent the number of cases. [file CNR2-7-e2004-s003.pdf]

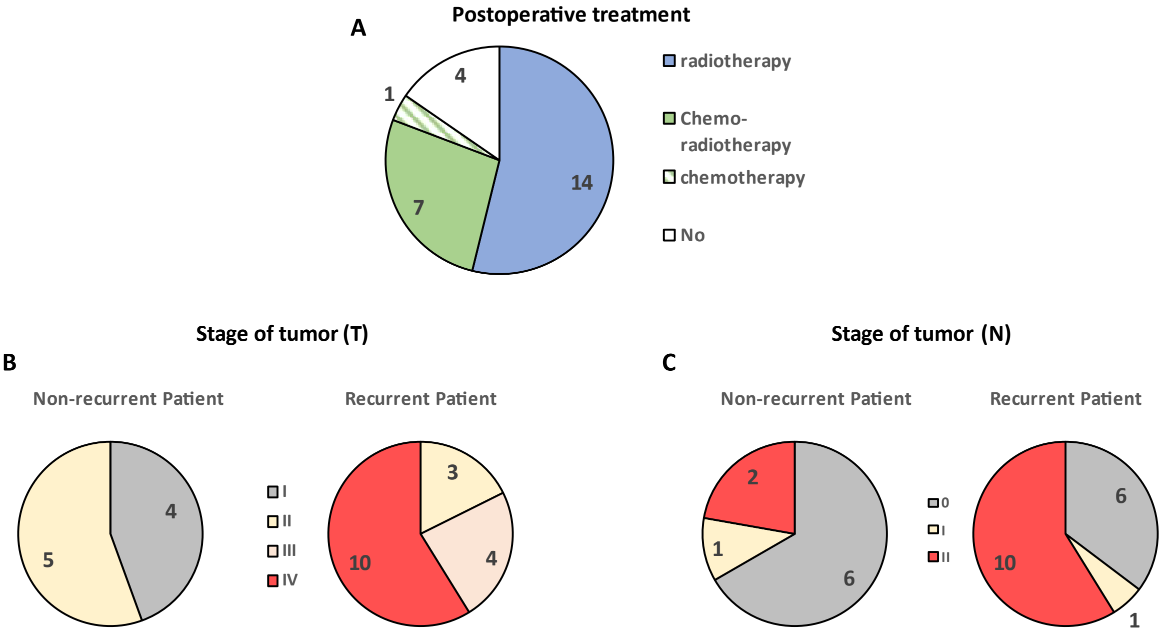

Supplemental Figure 1. Pie charts of patient characteristics in our cohort. A. Postoperative treatment data. B. Comparison of Stage of tumor (T) between Non-recurrent patients and recurrent patients C. Comparison of Stage of tumor (N) between Non-recurrent patients and recurrent patients. The numbers in the pie charts represent the number of cases.
