# Supplementary material for: Comparison of mutation landscapes of pretreatment versus recurrent squamous cell carcinoma of the oral cavity: The possible mechanism of resistance to standard treatment
Source: Cancer Rep (Hoboken). 2024 Mar 13;7(3):e2004. doi: 10.1002/cnr2.2004 (PMC10935893; doi:10.1002/cnr2.2004)
Supplement: Supplementary file 2 — Figure S2. Mutations in the OSCC patients in this study. (A) variant classification in the OSCC, Y‐axis indicates types of variant and X‐axis indicates numbers of variant. (B) Top 10 frequently mutated genes, Y‐axis indicates genes and X‐axis indicates numbers of variant. [file CNR2-7-e2004-s001.pdf]

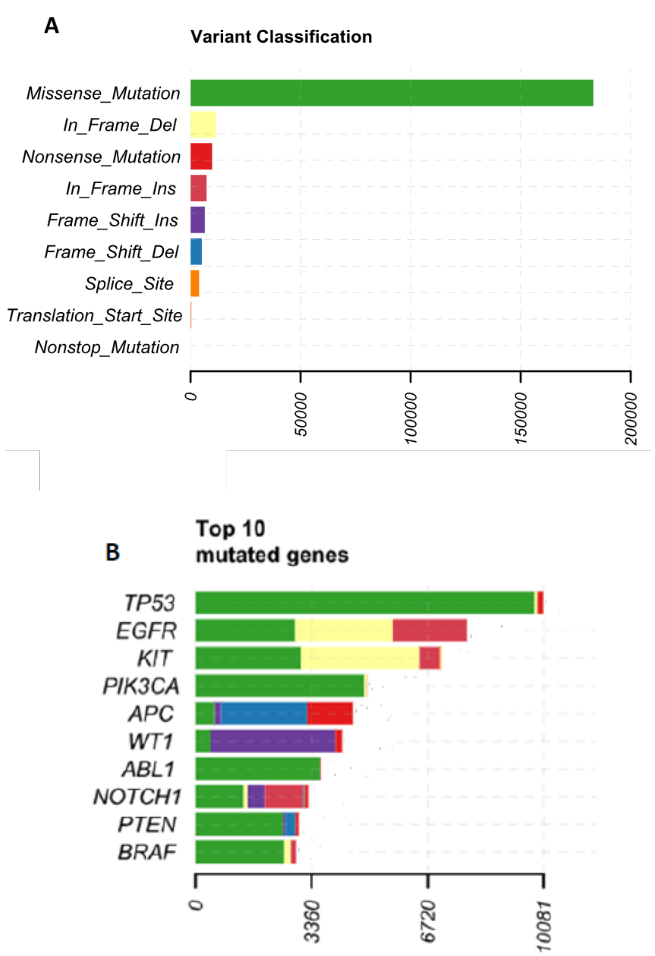

Supplemental Figure 2. Mutations in the OSCC patients in this study A. variant classification in the OSCC, Y-axis indicates types of variant and X-axis indicates numbers of variant. B. top 10 frequently mutated genes, Y-axis indicates genes and X-axis indicates numbers of variant.
