# Supplementary material for: Comparison of mutation landscapes of pretreatment versus recurrent squamous cell carcinoma of the oral cavity: The possible mechanism of resistance to standard treatment
Source: Cancer Rep (Hoboken). 2024 Mar 13;7(3):e2004. doi: 10.1002/cnr2.2004 (PMC10935893; doi:10.1002/cnr2.2004)
Supplement: Supplementary file 3 — Figure S3. Mutation landscape of OSCC patients in this study. Each row indicates the gene, and each column indicates the patient. The bars on the right‐hand side show the number of patients containing mutated genes in each row. Types of mutation are indicated in colors at the bottom of the figure. Colored bars on top of the figure indicate the group of OSCC patients. [file CNR2-7-e2004-s002.pdf]

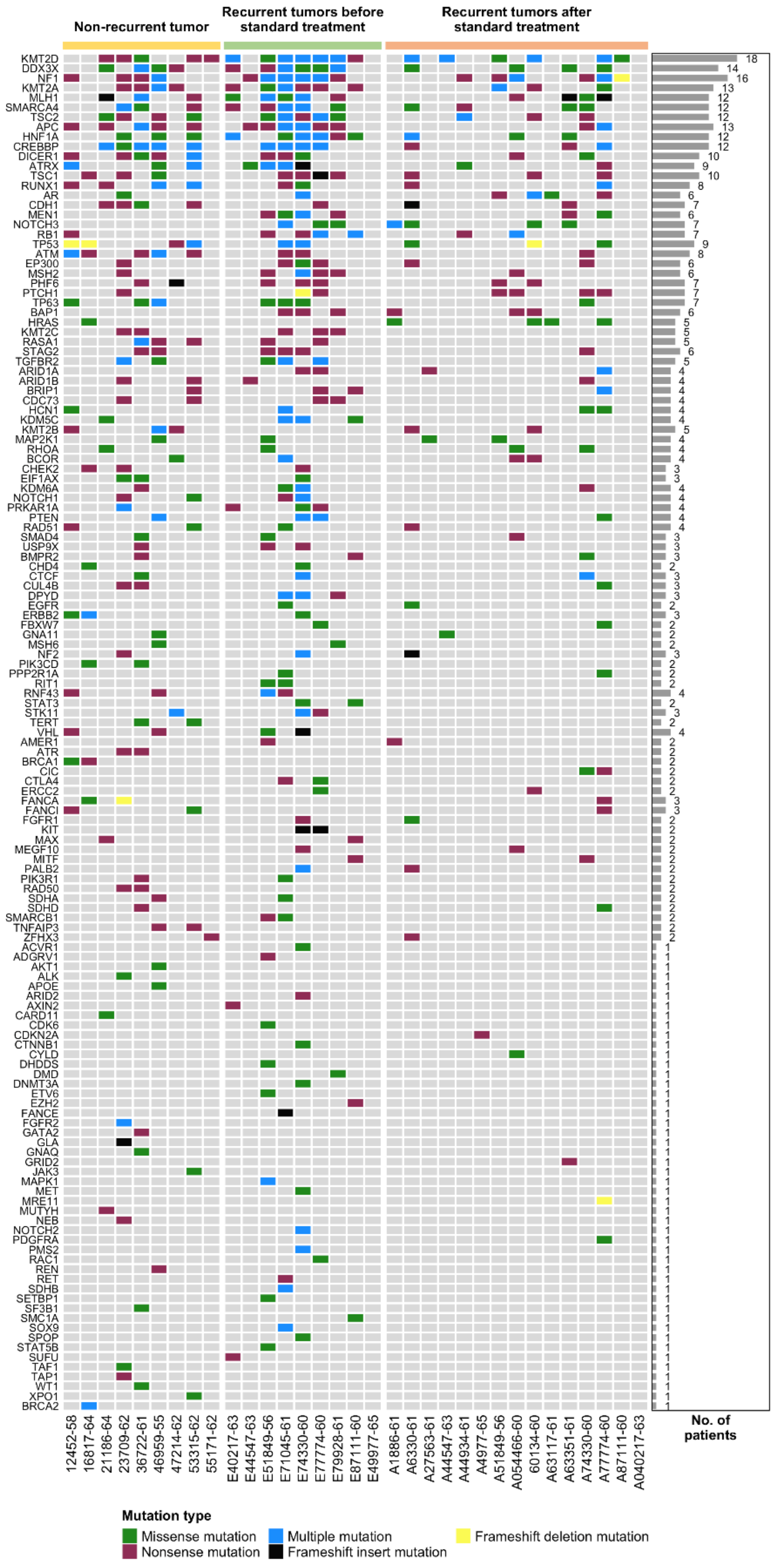

Supplemental Figure 3 Mutation landscape of OSCC patients in this study. Each row indicates the gene, and each column indicates the patient. The bars on the right-hand side show the number of patients containing mutated genes in each row. Types of mutation are indicated in colors at the bottom of the figure. Colored bars on top of the figure indicate the group of OSCC patients.
